# LCQS: an efficient lossless compression tool of quality scores with random access functionality

**DOI:** 10.1186/s12859-020-3428-7

**Published:** 2020-03-18

**Authors:** Jiabing Fu, Bixin Ke, Shoubin Dong

**Affiliations:** 1 School of Computer Science & Engineering, South China University of Technology, Wushan Road, Guangzhou, 510006 China; 2 Communication & Computer Network Lab of Guangdong, South China University of Technology, Wushan Road, Guangzhou, 510006 China

**Keywords:** Quality score, Lossless compression, Random access, Robust, Efficient, Parallelization, ZPAQ

## Abstract

**Background:**

Advanced sequencing machines dramatically speed up the generation of genomic data, which makes the demand of efficient compression of sequencing data extremely urgent and significant. As the most difficult part of the standard sequencing data format FASTQ, compression of the quality score has become a conundrum in the development of FASTQ compression. Existing lossless compressors of quality scores mainly utilize specific patterns generated by specific sequencer and complex context modeling techniques to solve the problem of low compression ratio. However, the main drawbacks of these compressors are the problem of weak robustness which means unstable or even unavailable results of sequencing files and the problem of slow compression speed. Meanwhile, some compressors attempt to construct a fine-grained index structure to solve the problem of slow random access decompression speed. However, they solve the problem at the sacrifice of compression speed and at the expense of large index files, which makes them inefficient and impractical. Therefore, an efficient lossless compressor of quality scores with strong robustness, high compression ratio, fast compression and random access decompression speed is urgently needed and of great significance.

**Results:**

In this paper, based on the idea of maximizing the use of hardware resources, LCQS, a lossless compression tool specialized for quality scores, was proposed. It consists of four sequential processing steps: partitioning, indexing, packing and parallelizing. Experimental results reveal that LCQS outperforms all the other state-of-the-art compressors on all criteria except for the compression speed on the dataset SRR1284073. Furthermore, LCQS presents strong robustness on all the test datasets, with its acceleration ratios of compression speed increasing by up to 29.1x, its file size reducing by up to 28.78%, and its random access decompression speed increasing by up to 2.1x. Additionally, LCQS also exhibits strong scalability. That is, the compression speed increases almost linearly as the size of input dataset increases.

**Conclusion:**

The ability to handle all different kinds of quality scores and superiority in compression ratio and compression speed make LCQS a high-efficient and advanced lossless quality score compressor, along with its strength of fast random access decompression. Our tool LCQS can be downloaded from https://github.com/SCUT-CCNL/LCQSand freely available for non-commercial usage.

## Background

Driven by the enormous scientific success of world-wide HGP (Human Genome Project), NGS (Next Generation Sequencing) has made tremendous progress in recent decades and thus enables high throughput of the production of the FASTQ files [1] at a low cost. Data storage and transmission become the main bottleneck of genomic data related analysis. As the affiliated sequencing error measurement of genomic sequence read, quality scores occupy at least 70% storage space of lossless compressed FASTQ file [2]. The high randomness caused by the noise of the quality score have made the compression of FASTQ low-efficient. However, quality scores in the FASTQ file play an indispensable role in many subsequent analyses (such as sequence alignment and variant calling) and thus cannot be discarded directly [3]. Therefore, the demand for a specialized and efficient lossless compressor for quality score becomes urgent and significant.

The dominant view of the current challenge of compression tools lies in that they should be able to compress large files in a short time and with a limit amount of memory. Therefore, the performance of a lossless compressor is determined by the following four criteria:

1. Compression Ratio: the less bits it uses to restore the original file, the better ratio it has;
2. Compression Speed: the less time it uses to transform the original file into compressed form, the better speed it has;
3. Decompression Speed: the less time it uses to restore the original file from its compressed form, the better speed it has;
4. Memory Usage: the less memory it uses to compress or decompress the original file, the better performance it has.

Consequently, improving these four criteria to the utmost becomes critical to developing an outstanding lossless compressor. However, these four criteria are not independent but mutually restrictive. Most existing lossless quality score compressors [4–6] adopt the design pattern of “sacrificing one for another” in the classic evaluating paradigm of “Compression Ratio, Compression Speed, Decompression Speed, and Memory usage”. For instance, some compressors try to sacrifice the compression ratio by applying a simple probability model to compress or decompress at a very high speed. Many compressors try to sacrifice the robustness by fixing the length of quality scores to improve the compression ratio. However, varied length quality scores are an essential part of many critical intermediate files (e.g., SAM format file [7]) generated during the variant calling procedure. The complicated probability model is applied to model quality scores accurately to improve the compression ratio at the sacrifice of compression speed. It is challenging to balance the four criteria, which makes the design of an efficient compressor almost impossible.

However, the current challenge proposed by the majority is a general challenge for any data compression problems and is only one kind of existing challenges. This broad understanding of the current challenge might not apply to the compression of specific data since challenge should vary from scene to scene in the context of quality scores which is the focus of our research. Quality score, as the measurement of the level of confidence of an individual sequenced base call, has its particular way of usage and is commonly used to act as backups in store for future’s specific look-up. Therefore, random access decompression of quality scores is more important than complete decompression. A new evaluating paradigm of “Compression Ratio, Compression Speed, Random Access Decompression Speed, and Memory usage” could be more appropriate for the lossless compression algorithm of quality score. This exclusive property of quality score compression makes it relatively easy to improve all the four criteria together due to sharply weakened impact of decompression speed, and more attention paid to optimize the other three criteria. A recent excellent lossless quality score compressor AQUa [8] adopts the new evaluating paradigm above and provides fast random access decompression support. However, huge sacrifices of compression speed and extra size of index files prevent it from practical usage. Meanwhile, AQUa can only handle quality score lines with the same length and has the drawback to dealing with the quality score with varied lengths. However, an advanced compression tool should possess the ability to compress any different forms of quality scores. The more input sources it can handle, the better robustness it has. Hence, a new evaluative criterion Robustness is fleshed out in the evaluative paradigm.

However, theoretically speaking, for an unknown genomic dataset, it is impossible to balance the five criteria of the new evaluative paradigm of “Robustness, Compression ratio, Compression speed, Random Access Decompression speed and Memory usage” at the same time since there is no free lunch. That is, reducing more redundancy on more various forms of quality score naturally needs more searching time and more memory in finding data redundancy. Therefore, we do not follow the line of the majority who focus on achieving better results based on as fewer memory resources as possible. That is, memory usage should not be as less as possible. On the contrary, memory usage should be utilized to the most as long as it does not become a performance bottleneck of other hardware resources. In the same vein, CPU should be utilized to the most to achieve higher parallelization. That is, we utilize the hardware resource to the utmost to improve compression performance to take full advantage of easy-access and well-developed hardware resources to deal with the “No Free Lunch” dilemma.

Based on the analysis above, we remove the memory usage criteria and propose an new lossless quality score compression algorithm evaluating paradigm of “Robustness, Compression Ratio, Compression Speed and Random Access Decompression Speed”. This paradigm is motivated by the idea of utilizing the hardware resource to the utmost. In this paper, we use the ratio of memory usage and CPU usage to guide the utilization of hardware resources. To sum up, under the condition that the ratio of memory usage and CPU usage is controlled to be less than a proper value, the current goal and challenge for quality score compression are to satisfy the following four criteria:

1. High Robustness: Whether the quality score’s length is varied or not, the quality score’s coding standard is varied or not, quality score’s species is human or not, the compression tool can compress any of them and obtain a stable compressed result;
2. High Compression Ratio: For any different kinds of quality scores, the compression tool can provide a competitive compression ratio;
3. High Compression Speed: For quality score file of small size (usually non-human genomic data), the compression tool can provide a competitive compression speed when compared with a state-of-the-art compression tool using an ordinary computer. For medium and large size quality score file, the compression tool possesses the property of high scalability and can provide a much more competitive compression speed when compared with other state-of-the-art compression tools through utilizing the more advanced hardware resource to the utmost;
4. High Random Access Decompression Speed: For compressed results of any size, fast and stable line-wise random access decompression and look-ups should be supported.

**Fig. 1:**
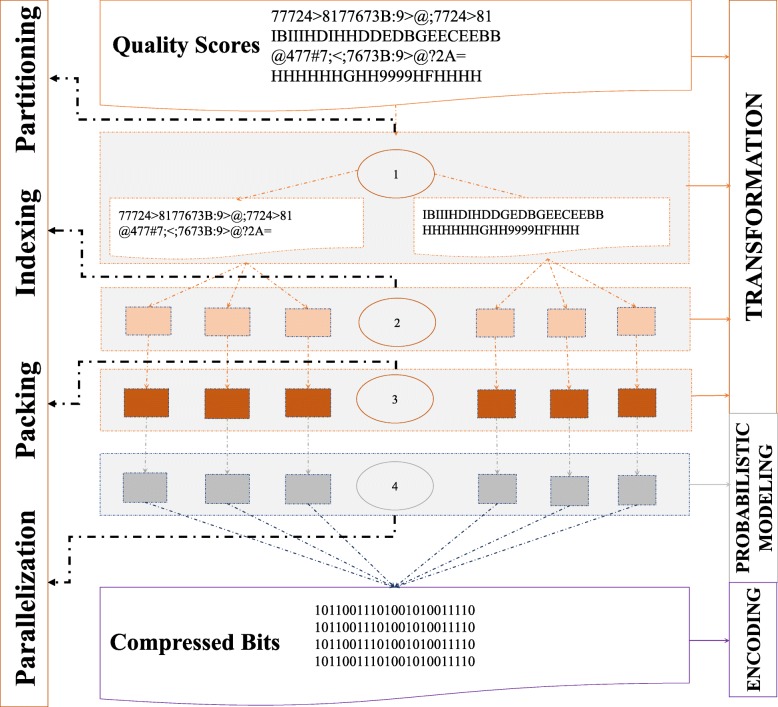
The framework of Proposed Lossless Compressor LCQS

In this paper, we aim at optimizing all the four criteria in the new evaluating paradigm at the same time as we can and designing an efficient lossless quality score compressor with random access decompression functionality. Our compressor LCQS includes four sequential processing steps: partitioning, indexing, packing, and parallelizing. Our framework is illustrated in Fig. 1. Regarding robustness, Fig. 1 shows that we proposed and applied several general prior observations but prohibited any specific priors. Regarding compression ratio, a robust data partition method (see step 1 in Fig. 1) based on general prior is proposed to capture different patterns of quality score content within a file. Furthermore, a complicated context mixing probabilistic modeling algorithm (see step 4 in Fig. 1) is used to capture the underlined pattern accurately to the utmost. Regarding compression speed, an adaptive quality score packing algorithm (see step 3 in Fig. 1) is proposed to reduce the content needed to be modeled. Furthermore, a parallelization strategy based on SIMD technique (see step 4 in Fig. 1) is used to optimize existing classical compression library libzpaq to speed up the modeling procedure of each piece of content. Regarding random access decompression speed, a light-weight index design (see step 2 in Fig. 1) is proposed to support fast and stable line-wise quality score random access decompression. More details about the four steps are discussed in the next section.

## Implementation

In this paper, robustness is the primary objective and is emphasized at the very beginning phase of algorithm design since we aim to design a practical and general compressor. The robustness of compressors lays based on general prior and thus the selection of general prior becomes crucial. This paper holds the idea that a good selection of general priors should be dated back to its origin. Back to the generation process of quality scores, we find that the content of the quality score is determined by two factors:

1. the original probability confidence level generated by different sequencing machines;
2. the storage format standard established by different communities;

Therefore, general priors should exhibit invariability among different sequencing machines and different communities. Based on the above analyses, this paper selects three general priors:

1. The ASCII values of quality score lie in the interval ranging from 33 to 104 due to the data format standard;
2. Quality score value follows very uneven distribution due to the effectiveness of sequencing machine;
3. Quality scores are generated by a mixture of different source distributions due to the inevitable disturbance produced during the process of sequencing machines estimating the sequence base’s confidence [6].

As is shown in Fig. 1, data compression can be viewed as a combination of various transformations, probabilistic modeling techniques and encoding strategies [9]. Data transformations and probabilistic modeling become the key to the optimization of compression methods since encoding techniques are mature and perform very well with theoretical guarantee. In this paper, all the data transformations and models proposed are designed based on only the three general priors and are discussed in detail in Fig. 1. “Quality score line partition method” section is designed to improve the compression ratio. “Light-Weight index design method” section is incorporated to support random access decompression functionality. “Adaptive k-mer packing method” and “Parallelization method for libzpaq using SIMD technique” sections are two optimized procedures for compression speed.

### Quality score line partition method

As is shown in Fig. 1, quality score lines within one quality score file might exhibit differences, which validates the third general prior mentioned before. Some lossless compressors [6, 10] attempt to apply different clustering methods to split the original fixed length quality score into several blocks. However, there are two main drawbacks:

1. Weak robustness. It does not work on varied length quality scores. Strong specific assumption is made to cluster better, which reduces the compressors’ robustness;
2. Low compression/decompression speed. Too much time is used to cluster the quality scores as accurately as possible. However, it might not be a wise trade-off to achieve slight improvements in the compression ratio by wasting too much time. As is noted by AQUa [8], multi-pass quality score compression method is not suitable for real-time quality score compression. Furthermore, single-pass compressors can minimize the latency between sequencing and genomic data analysis. Therefore, a robust and coarse partition method is appropriate.

In this paper, quality score line is represented by k-mers (refer to the substrings of length k) to ensure compression ratio and robustness since k-mer contains high-order context information and can be used to represent varied-length quality score line. With a view to both simple and validity, quality score lines here are represented by k-mers. Meanwhile, quality score lines tend to be similar when they share high-frequency k-mers and vice versa. Therefore, higher weight should be assigned to high-frequency k-mers and low-frequency k-mers should be assigned lower weight. To speed up the weight assignment process, we do weight assignments by utilizing only a subset of the dataset since we assume that the subset and whole dataset follow the same distribution. Specifically, the first M lines (Default 10^5^ lines) of the dataset were analyzed. The information of each k-mer’s occurrences and the total occurrences of all k-mers are then collected. The weight of one k-mer is assigned as the ratio of its occurrences to the total occurrences of all k-mers. The weight of each quality score line equals to the ratio of the sum of all its k-mers’ weights to the number of the k-mers within it. The maximum weight among the M sampling quality score line is obtained and is used to normalize all the other quality score line weights. Finally, quality score would be partitioned into several parts according to their different line weights. In this paper, only two clusters are generated since it is good enough to achieve a better balance between time and space. Step 1 (see Fig. 1) is the quality score line partition method and is illustrated in Algorithm 1 in Additional file 1.

### Light-Weight index design method

**Fig. 2:**
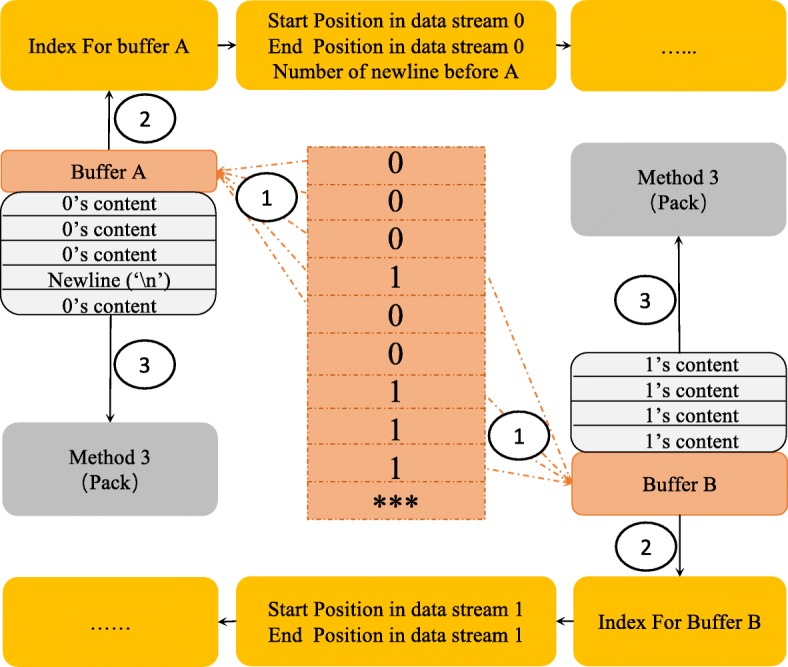
The Procedure of Light-weight Index Method: Step 2

As is shown in Fig. 2, once data streams 0 and 1 are obtained, data blocks to be compressed would be generated. The construction work of the index should be made and completed before feeding them into the next step to support line-wise random access decompression function. Two fixed-size buffers (A and B) are maintained to partition the two data streams into data blocks. For buffer A, the content of one quality score line would be copied into buffer A if it belongs to data stream 0. Meanwhile, a newline would be copied into buffer A to keep track of the order information if it belongs to data stream 1. For buffer B, the content of one quality score line would be copied into buffer B if it belongs to data stream 1. Furthermore, the content of one quality score line would be discarded if it belongs to data stream 0.

In order to achieve load balance, the buffer would be emptied and fed into the next step only when the size of the buffer exceeds a predefined threshold. Meanwhile, in order to keep the performance of random access decompression much more stable, buffer A would be forced to be emptied when buffer B is emptied for consecutive three times. Once two buffers are emptied for the next packing phase, the index information would be recorded. Regarding buffer A, the start position, the end position and the number of newline before A are recorded one by one in the sequential order of data stream 0. Regarding buffer B, only the start position and end position are recorded one by one in the sequential order of data stream 1 since the third entry can be inferred from the index information of A. Given a line-wise random access range such as [a, b], all the compressed data blocks of stream 0 and 1 whose range is overlapped with [a, b] would be extracted through looking up the index range information of the compressed data block. Once all needed compressed data blocks are decompressed and merged, the result content of range [a, b] is obtained. Normally, most of the range interval of the random access operation would not exceed the range of one data block and the number of data blocks needed to be decompressed would not exceed four (one A and three B). In the worst situation, the number of data blocks needed to be decompressed would not exceed eight (two A and six B) when the range interval of random access operation contains the boundary of two continuous block A.

### Adaptive k-mer packing method

As is mentioned in the introduction section, a complicated context mixing probabilistic modeling algorithm ZPAQ would be applied to capture the underlined pattern of quality score accurately. To reduce the negative effect of compression speed, we need to improve the compression speed to the best of our ability. In a nutshell, two solutions are proposed:

1. reducing the content that needs to be modeled;
2. reducing the time used to model each unit of the content.

As is shown in Fig. 1, the first one discussed in this section is usually completed in the data transformation phase while the second one as the focus of next section is usually optimized in the probabilistic modeling phase. Inspired by Bonfield and Mahoney [4] and the first general prior, packing techniques are applied to reduce the content that needs to be accurately modeled. Specifically, quality score k-mers in which each quality score value ranges from 33 to 104 are losslessly transformed into a single-digit number ranging from 1 to 255 through a one-to-one mapping rule. Nevertheless, this paper adaptively packs k-mer based on real distribution of quality score value. It is different from Mahoney’s method which applies the same fixed mapping rule to all different kinds of quality score datasets exhibiting different distributions. Adaptive k-mer packing is shown in Fig. 3 and is implemented as follows:

1. Obtaining the quality score value which has the highest occurrences and denoting it as C. As is shown in Fig. 3, the mapping rule is fixed once C is obtained. The reason why we choose the quality score with the highest occurrences as the boundary of transformation interval lies in that it possesses the highest possibility of forming k-mer as many as possible through combination with other quality score values. The more k-mer satisfies the transformation conditions, the more content it can reduce;

**Fig. 3:**
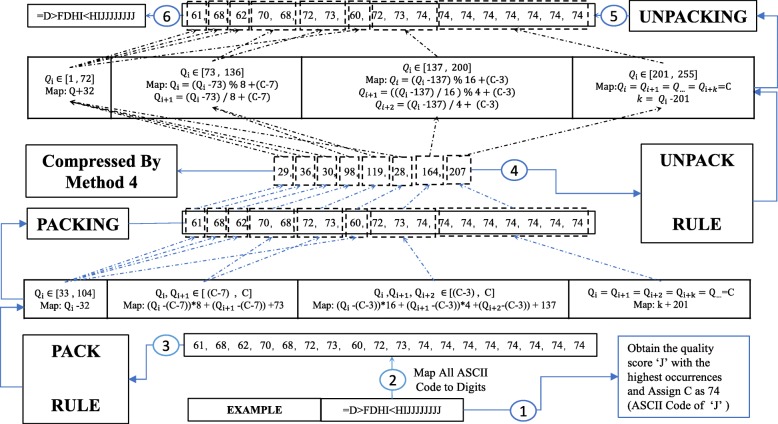
The Procedure of Adaptive k-mer Packing Method: Step 3
2. Grouping and mapping of the quality score. As is shown in Fig. 3, for each quality score, the priority of grouping and mapping of k-mers increases from left to right (1⇒255). Details about the packing procedure of Fig. 3 can be seen in the implementation details of the adaptive k-mer packing method which is presented in the Additional file 2. The whole mapping procedures are visualized in detail in Fig. 3. Meanwhile, an example is provided to understand the adaptive k-mer mapping method better. The mapping is one-to-one so quality score value can be easily unpacked in a reverse way.

### Parallelization method for libzpaq using SIMD technique

Complicated probabilistic modeling means slow modeling speed. To further improve the modeling speed and reduce the modeled content discussed in “Adaptive k-mer packing method” section, this paper attempts to use SIMD and Multithreading techniques to shorten the time of modeling each unit content.

Libzpaq [11] was born in 2009 and written by Matt Mahoney. It is a state-of-the-art backend open-source compression library and is widely used all around the world. It possesses excellent performance on the compression ratio at the expense of slow compression speed. Therefore, code optimization is always the focus of libzpaq compression community. Libzpaq’s compression speed has been highly optimized and dramatically improved in continuously iterated versions by utilizing different kinds of accelerating techniques. However, there is still some distance away from practical usage. Once its compression speed becomes acceptable in practical usage, existing excellent quality score compressors (not limited to quality score compression) would become acceptable since many compressors [4, 5, 10, 12] use libzpaq as their backend compressor. Thus, it is of great importance to optimize libzpaq’s performance of compression speed.

This paper analyzes all the points which have the potential to speed up to optimize libzpaq. From the perspective of theoretical analysis, the predictor of libzpaq should be very time-consuming since libzpaq’s main work is to accurately predict the compressed source using highly complex context mixing probabilistic model to achieve excellent performance on compression ratio. From the perspective of experimental analysis, the libzpaq library’s predictor module is always time-consuming, which validates our assumption. After further investigating the predictor module, this paper finally selects two submodules (Update and Predict Module of Predictor) of libzpaq as our primary optimized points and uses SIMD techniques to rewrite the libzpaq using C++ programing language. Currently, libzpaq compression library has JIT and NON-JIT versions. Although only JIT version of libzpaq is used in our compressor LCQS, we optimize libzpaq library for both versions to make it much more universal for uses for other purposes by other compressors. Furthermore, we not only incorporate the optimized libzpaq code into LCQS but also pack it into an independent component which can be easily called in other compressors.

## Results

This section describes experimental setups in detail and validates the effectiveness of our proposed lossless quality score compressor LCQS. We compare our tool with three state-of-the-art compressors on recognized benchmark datasets in terms of the following four new criteria of efficiency: Robustness, Compression Ratio, Compression Speed and Random Access Decompression Speed. Meanwhile, as an independent component, compression library libzpaq is also tested and compared.

### Benchmark datasets selection

Datasets with different statistical properties would result in a strong bias on the performance of the compressors. Thus, much attention should be paid to choose test datasets to evaluate the effectiveness of proposed compressors. With huge demand and rapid development of genomic information compression, the standardization of genomic data benchmark becomes urgent. Currently, the MPEG HTS compression working group, is aware of the urgency and is building up the genomic data benchmark. Nevertheless, as is noted by Numanagic et al. [9], the size of the datasets compiled by MPEG HTS compression working group consists of approximately 4 TB and is expecting a rise in the future. Numanagc et al. [9] establishes a new acceptable and reasonable benchmark from the MPEG datasets. We choose it as our test benchmark. Besides, to test the performance of compressing large genomic files, we also collect high coverage reads for NA12878 from the public website [13]. Only the quality score part of the benchmark FASTQ datasets samples [13, 14] is extracted, and thus their sizes are different from their original FASTQ files. For the datasets from 1_01 to 5_02, quality scores are extracted directly from the corresponding FASTQ file. Besides, we extract the quality scores of the eight large datasets (ERR091571_1.fastq 47 GB, ERR091571_2.fastq 47 GB, ERR091572_1.fastq 47 GB, ERR091572_2.fastq 47 GB, ERR091573_1.fastq 47 GB, ERR091573_2.fastq 47 GB, ERR091574_1.fastq 49 GB, ERR091574_2.fastq 49 GB) from the public website [13]. Then we preprocess the eight large datasets into the quality score dataset ERR09157 and denotes ERR09157 as 6_01. Detailed information about the obtained quality score datasets is presented in Table 1. It is clear from Table 1 that the test datasets have good diversity since they consist of datasets with different species, different technologies, different sequencing depths, different lengths and different sizes. Therefore, comparing results tested on that benchmark would be representative and convincing.

**Table 1:** Detailed Descriptions of Test Quality Score Datasets

Code	Filename(quality score only)	Organism	Technology	Coverage	Length	Size(MB)
1_01	SRR554369_1	P.Aeruginosa	Illumina GAIIx	50x	100	160
1_02	SRR554369_2	P.Aeruginosa	Illumina GAIIx	50x	100	160
2_01	MH0001_081026_clean.1	H.Sapiensgut	Illumina GAII	Unknown	44	500
2_02	MH0001_081026_clean.2	H.Sapiensgut	Illumina GAII	Unknown	44	500
3_01	SRR1284073	E.Coli	PacBio	140x	Varied	620
4_01	SRR327342_1	S.Cerevisiae	Illumina GAII	175x	75	918
4_02	SRR327342_2	S.Cerevisiae	Illumina GAII	175x	75	1090
5_02	SRR870667_2	T.Cacao	Illumina GAIIx	35x	74	4952
5_01	SRR870667_1	T.Cacao	Illumina GAIIx	35x	74	7197
6_01	ERR09157	Human	Illumina	Unknown	101	166,142

### Benchmark compressors selection

To better evaluate the performance of our proposed lossless quality score compressor LCQS, both general and specialized lossless benchmark compressors are selected. Concerning general benchmark compressors, it is easy to make choices and two excellent compressors (7-zip and Gzip) are selected. Concerning specialized benchmark compressors, it is difficult to make choices. Since our compressor is in a lossless manner, the related tool on specialized quality score compression is rare, which makes the work of choosing benchmark compressors difficult. Fortunately, quality score is one part of FASTQ file and many FASTQ compressors integrated the functionality of quality score compression in recent decades. However, without a deep understanding of the source code of the compressor, a simple calculation of separate and visible compressed results (e.g. Fastqz [4], Scalce [15]) would produce a wrong or bias compression ratio. Besides, the results of compression speed of quality score in FASTQ cannot be collected. Based on the above considerations, all FASTQ compressors are excluded. This paper chooses AQUa [8] as the specialized benchmark compressor to better evaluate the compression performance and random access decompression functionality under the new evaluating paradigm since AQUa is state-of-the-art and the only one that can provide random access decompression support.

Detailed information about the comparison compressors can be seen in Table 2. All experiments are tested on the same Linux server (Intel(R) CPU E5-2670 @ 2.60GHz, 16 CPU cores). The cache is cleared before every experiment test to avoid the effect caused the caching technique of the operating system.

**Table 2:** Detailed Descriptions of Benchmark Compressors

Compressors	Parameters	Source URLs
LCQS	k=4, α= 0.1 (they are defined in step 1)	https://github.com/SCUT-CCNL/LCQS
AQUa	windowsize=1, cabacgrouping=10485760	https://github.com/tparidae/AQUa
7-zip	-mx9	https://www.7-zip.org/
Gzip	-9	https://www.gnu.org/software/gzip/

### Benchmark criteria selection

To evaluate the performance of our proposed lossless compressor LCQS more comprehensively, as is mentioned in the introduction part, four criteria named Robustness, Compression Ratio, Compression Speed and Random Access Decompression Speed are chosen as the benchmark criteria in this paper. To accurately quantify the criteria, we redefine these four criteria clearly as follows:

1. Robustness is calculated using the ratio of the number of the datasets that compressor can both compress and random access decompress to the number of benchmark datasets.
2. Compression Ratio is calculated using the ratio of the size of megabytes of the compressed datasets (With the index used for random access excluded) to the size of megabytes of the original benchmark dataset. Since Gzip and 7-zip do not support random access operation and the index is used only when random access decompression operation is carried out, the size of the metadata index used for random access decompression is excluded when calculating compression ratio. The size of the metadata index is excluded here and would be in store for assisting the evaluation of the performance of random access decompression to evaluate the performance of benchmark compressors much more general and fairer.
3. Compression Speed is calculated using the ratio of the size of megabytes of the original benchmark dataset to the time of seconds used to compress the dataset.
4. Random Access Decompression Speed is calculated using the ratio of the number of thousand lines of the given random access range interval to the time of seconds used to random access decompress it.

### Comparison results among benchmark compressors

The four criteria mentioned in “Benchmark criteria selection” section are evaluated in detail one by one in this section. The best compression results for each dataset in all the following tables are bolded and “-” mean that this file cannot be compressed or random access decompressed by that compressor.

#### Performance of robustness and compression ratio

Table 3 shows the compression ratio results which compared LCQS with the other three compressors. Regarding robustness, AQUa [8] performs the worst. LCQS, together with Gzip and 7-zip, perform the best and can compress all the benchmark datasets. Regarding the compression ratio, LCQS outperforms all the datasets with an obvious advantage and file sizes have reduced by up to 18.92%, 16.68% and 28.78% respectively when benchmarking LCQS against AQUa, 7-zip at best compression mode and Gzip at best compression mode. Therefore, LCQS performs the best on both robustness and compression ratio and thus its effectiveness is validated. Detailed comparison results are presented in Table 3.

**Table 3:** Comparison Results of Compression Ratio

Datasets	Compression Ratio				LCQS File Size Reduction Versus (%)		
	LCQS	AQUa	7-zip best	Gzip best	AQUa	7-zip best	Gzip best
1_01	**3.4388**	2.9726	2.9351	2.5884	13.56	14.65	24.73
1_02	**3.3241**	2.9296	2.8668	2.5365	11.87	13.76	23.69
2_01	**3.5023**	3.1762	3.1570	2.8401	9.31	9.86	18.91
2_02	**2.4592**	2.1817	2.2387	2.0756	11.29	8.97	15.60
3_01	**2.5911**	-	2.3159	2.1041	-	10.62	18.80
4_01	**2.7909**	2.5730	2.5093	2.2453	7.81	10.09	19.55
4_02	**2.5749**	2.3483	2.3099	2.0933	8.80	10.29	18.70
5_02	**2.8598**	2.5795	2.5400	2.2735	9.80	11.18	20.50
5_01	**3.2533**	2.8602	2.8276	2.4974	12.08	13.09	23.23
6_01	**3.9660**	3.2156	3.3046	2.8245	**18.92**	**16.68**	**28.78**

#### Performance of compression speed

Table 4 shows the compression speed results which compared LCQS with the other three compressors. Regarding the compression speed, LCQS outperforms almost all benchmark datasets except the result on dataset 3_01 compressed by Gzip. The acceleration ratios have increased by up to 29.1x, 8.4x, and 4.3x when benchmarking LCQS against AQUa, 7-zip at best compression mode and Gzip at best compression mode respectively. Besides, LCQS’s compression speed tends to scale linearly with the increasing datasets to be compressed due to its high parallelization characteristic. Our test computer has 16 hyper-threaded cores and LCQS can occupy almost all the whole 3200% CPU. To be concluded, LCQS has superior advantages over compression speed and is expected to be applied in real practical scenarios of large datasets due to its high scalability.

**Table 4:** Comparison Results of Compression Speed

Datasets	Compression Speed (MB/s)				LCQS Accelerating Ratio Versus%		
	LCQS	AQUa	7-zip best	Gzip best	AQUa	7-zip best	Gzip best
1_01	**2.32**	0.31	1.09	2.25	648%	113%	3%
1_02	**2.29**	0.31	1.04	1.79	639%	120%	28%
2_01	**5.15**	0.30	1.35	1.58	1617%	281%	226%
2_02	**4.55**	0.26	1.34	1.88	1650%	240%	142%
3_01	4.63	-	0.98	**5.84**	-	372%	-21%
4_01	**5.92**	0.31	0.99	2.39	1810%	498%	148%
4_02	**6.12**	0.31	0.88	3.84	1874%	595%	59%
5_02	**6.33**	0.31	1.00	2.03	1942%	533%	212%
5_01	**9.29**	0.31	0.99	1.75	2897%	**838%**	**431%**
6_01	**9.63**	0.32	1.09	2.28	**2909%**	783%	322%

Table 5 shows that all the ratio of Memory Usage(GB) to CPU Usage(Thread) (hereinafter called M/C in Table 5) of LCQS can be controlled to be less than 0.54 and about 0.45 on average, which can be satisfied by almost all ordinary computers. That is, memory usage of LCQS will not influence LCQS’s high scalability and thus makes LCQS a practical quality score compression tool for almost all computing platforms. Although the high scalability of LCQS has already outperformed all other compressors, it has not yet shown its best performance due to the hardware limit. The compression speed of LCQS would be boosted when more CPU cores are available. On the contrary, the other three compared compressors exhibit relatively low efficiency of utilizing the hardware resources (here, CPU and memory) when compared with LCQS, especially for Gzip, which results in the fall of compression performance.

**Table 5:** Comparison Results of CPU Usage and MEMORY Usage

Datasets	AVERAGE CPU USAGE(%)				AVERAGE MEMORY USAGE(GB)				M/C
	LCQS	AQUa	7-zip best	Gzip best	LCQS	AQUa	7-zip best	Gzip best	LCQS
1_01	**400**	104	176	99	1.32	0.57	0.58	**0.0016**	**0.33**
1_02	**382**	104	175	99	1.26	0.57	0.56	**0.0017**	0.33
2_01	**900**	105	180	100	3.82	0.6	0.63	**0.0016**	0.42
2_02	**1036**	105	175	99	4.6	0.61	0.64	**0.0016**	0.44
3_01	**1298**	-	157	99	6.12	-	0.65	**0.0017**	0.47
4_01	**1635**	105	168	99	7.52	0.63	0.65	**0.0016**	0.46
4_02	**2030**	105	165	99	9.26	0.63	0.66	**0.0016**	0.46
5_02	**2932**	104	173	100	15.6	0.63	0.67	**0.0016**	0.53
5_01	**2932**	105	177	100	14.37	0.66	0.67	**0.0016**	0.49
6_01	**3161**	105	172	100	16.99	0.66	0.67	**0.0016**	0.54

#### Performance of random access decompression speed

Table 6 shows the results of random decompression speed and the extra size of the metadata index of the original file. The specific interval for each dataset is randomly generated for interval sizes ranging from 40000 to 160000. Then, random access decompression operation is applied on the specific interval.

**Table 6:** Comparison of Random Access Decompression Functionality

Datasets	Random Access Decompression Speed (Thousand lines / s)						Extra index size (%)	
	LCQS			AQUa			LCQS	AQUa
	40000	80000	160000	40000	80000	160000		
1_01	**0.65**	**1.27**	**2.67**	-	-	-	**0**	40.73
1_02	**0.63**	**1.21**	**2.42**	-	-	-	**0**	40.73
2_01	**0.55**	**1.07**	**2.11**	0.53	0.83	-	**0**	92.94
2_02	**0.53**	**1.03**	**2.05**	0.24	0.47	-	**0**	93.46
3_01	**0.59**	**1.00**	**1.63**	-	-	-	**0**	-
4_01	**0.67**	**1.31**	**2.29**	0.33	0.75	-	**0**	65.85
4_02	**0.61**	**1.16**	**2.32**	0.47	0.61	-	**0**	55.7
5_02	**0.45**	**0.94**	**1.90**	0.35	0.78	-	**0**	56.62
5_01	**0.63**	**1.23**	**2.29**	0.26	0.40	-	**0**	39.18
6_01	**0.52**	**1.05**	**1.44**	0.40	0.65	-	**0**	41.79
Average	**0.58**	**1.13**	**2.11**	0.37	0.64	-	**0**	58.56

Concerning the time needed to random access to the given lines, our proposed LCQS outperforms all the ten benchmark datasets and exhibits high and stable performance when compared with AQUa. Regarding the random access decompression speed of thousand lines per second, our LCQS outperforms all the ten benchmark datasets and the acceleration ratios have increased by up to 1.4x and 2.1x when benchmarking LCQS against AQUa at the range interval of 40000 and 80000 respectively. Furthermore, LCQS exhibits strong scalability since the speed tends to scale almost linearly with the increasing range interval. Regarding the extra file size introduced by achieving random access decompression function, LCQS does not need any extra index size since the light-weight index occupies only several bytes’ space and has already been packed into the compressed file.

On the contrary, AQUa designs a fine-grained and uncompressed index structure to enable ultrafast random access to the compressed file. Therefore, AQUa needs a large index file whose size ranges from 58.56% (average) to 93.46% (worst case) of the size of its uncompressed file. Concerning the robustness of random access decompression, different from AQUa which fails to apply operations with some range intervals and some benchmark datasets, LCQS can complete all random access operations with any range intervals on all benchmark datasets.

### Optimization of libzpaq library using sIMD technique

Table 7 shows the result which compared optimized libzpaq using SIMD technique LCQS with the original libzpaq. As is mentioned in “Parallelization method for libzpaq using SIMD technique” section, two main versions of JIT and NON-JIT are both optimized using the same SIMD technique. Our optimized library libzpaq outperforms both original JIT and NON-JIT version and makes some big improvements on the compression speed by up to 22.35% and 22.95% respectively. Detailed information can be seen in Table 7.

**Table 7:** Optimization Result of Libzpaq Library Using SIMD

Datasets	Improvements (%)	
	JIT	NON-JIT
1_01	**22.35**	18.63
1_02	20.33	21.55
2_01	16.15	16.43
2_02	16.35	16.17
3_01	16.17	19.17
4_01	16.75	19.96
4_02	16.52	19.16
5_02	12.27	19.47
5_01	15.96	**22.95**

## Conclusions

Tremendous progress of NGS in recent decades enables high throughput of the production of the FASTQ files. However, it also poses a big challenge to the existing lossless quality score compression tools. Therefore, LCQS, as an efficient lossless compression method of quality scores with random access functionality, is proposed in this paper.

The performance of LCQS was evaluated on ten benchmark real-world quality score datasets. Experimental results reveal that our compressor LCQS outperforms all compared compressors on all criteria except for the compression speed on the dataset SRR1284073. LCQS also exhibits the strongest scalability and thus is an efficient lossless compressor for practical usage. Meanwhile, an independent optimized backend compression library is developed and can be easily applied to boost the existing compression tools of quality score or bioinformatics-related data.

For future work, we attempt to improve the compression ratio by incorporating the existing deep learning techniques to capture the complex context information of quality scores and investigate the possibility of speed-up of libzpaq by utilizing GPU hardware. Besides, we would try to redesign and apply our quality score (In FASTQ format files) compressor LCQS for the varied length quality scores in SAM format file.

## Availability and requirements

Project name: LCQS Project website: https://github.com/SCUT-CCNL/LCQSOperating systems: Linux Programming language: C/C++ Other requirements: GCC compiler (Version 4.9+ is better)License: The MIT License Any restrictions to use by non-academics: For commercial use, please contact the authors.

## Supplementary information

**Additional file 1** The procedure of quality score line partition: step 1.

**Additional file 2** Implementation details of adaptive k-mer packing method.

## Abbreviations

CPU: Central Processing Unit
GB: GigaByte
GPU: Graphic Processing Unit
HGP: Human Genome Project
HTS: High-throughput Sequencing
JIT: Just-In-Time
MB: MegaByte
MPEG: Moving Picture Experts Group
NGS: Next Generation Sequencing
SAM: Sequence Alignment Map
SIMD: Single instruction, multiple data

## References

[1] FASTQ File Format. https://en.wikipedia.org/wiki/FASTQ_format. Accessed 10 Sept 2018.

[2] Hernaez M, Ochoa I, Weissman T, Bilgin A (2016). A cluster-based approach to compression of quality scores. Proceedings of Data Compression Conference.

[3] Ochoa I, Hernaez M, Goldfeder R, Weissman T, Ashley E (2017). Effect of lossy compression of quality scores on variant calling. Brief Bioinform.

[4] Bonfield JK, Mahoney MV (2013). Compression of fastq and sam format sequencing data. PloS ONE.

[5] Nicolae M, Pathak S, Rajasekaran S (2015). Lfqc: a lossless compression algorithm for fastq files. Bioinformatics.

[6] Hernaez M, Ochoa I, Rao M, Ganesan K, Weissmans T (2015). Qvz: lossy compression of quality values. Bioinformatics.

[7] SAM file format. https://en.wikipedia.org/wiki/SAM_(file_format). Accessed 10 Sept 2018.

[8] Paridaens T, Van Wallendael G, De Neve W, Lambert P (2018). Aqua: an adaptive framework for compression of sequencing quality scores with random access functionality. Bioinformatics.

[9] Numanagić I, Bonfield JK, Hach F (2016). Comparison of high-throughput sequencing data compression tools. Nat Methods.

[10] Fu JB, Ma YC, Ke BX, Dong SB. Proceedings of Bioinformatics and Biomedicine In: Bilgin A, et al., editors. Shenzhen: IEEE: 2016. p. 864–9.

[11] ZPAQ. http://mattmahoney.net/dc/zpaq.html. Accessed 10 Sept 2018.

[12] Huang ZA, Wen Z, Deng Q, Chu Y, Sun Y, Zhu Z (2017). Lw-fqzip 2: a parallelized reference-based compression of fastq files. BMC Bioinformatics.

[13] Dataset Description. http://smash.cs.berkeley.edu/datasets.html. Accessed 10 Sept 2018.

[14] Dataset Description. https://github.com/sfu-compbio/compression-benchmark/blob/master/samples.md. Accessed 10 Sept 2018.

[15] Faraz H, Ibrahim N, Can A, S Cenk S (2012). Scalce: boosting sequence compression algorithms using locally consistent encoding. Bioinformatics.

