# Supplementary material for: LCQS: an efficient lossless compression tool of quality scores with random access functionality
Source: BMC Bioinformatics. 2020 Mar 18;21:109. doi: 10.1186/s12859-020-3428-7 (PMC7079445; doi:10.1186/s12859-020-3428-7)
Supplement: Supplementary file 1 — Additional file 1 The procedure of quality score line partition: step 1. [file 12859_2020_3428_MOESM1_ESM.pdf]

Algorithm 1 Quality Score Line Partition Method

Input:

File Q with N quality score lines, Q^N;
Predefined number parameter of sampling quality score lines of input file Q, M(Deault 10^5);
Predefined size parameter of substrings of quality scores within one quality score line, k;
Predefined partition threhold, α;// Data are partitioned based on this threhold

Output:

Occurrence frequency table of substrings of length k , F ;// F(q) means the frequency of string q
Weight table of each entry in Frequency table F, S; // S(q) means the weight of string q
Output quality score stream 0, C_0;
Output quality score stream 1, C_1;

```
// Note*: All indexs shown in this algorithm start from 1, not 0.

// Phase 1: Collect statistical information among the first M sampling quality score lines .
Count_F ⇐ 0 // Record the total occurrence frequency of all k-mers
for i = 1 to M do
    len = length(Q_i) // Q_i means the ith line in Q^N. length(Q_i) means the length of Q_i.
    for j = 1 to (len − k + 1) do
        if Q_i[j,j+k-1] not in F // Q_i[m, n] means the substring of Q_i from index m to n then
            F[Q_i[j, j + k − 1]] ⇐ 1
        else
            F[Q_i[j, j + k − 1]] ⇐ F[Q_i[j, j + k − 1]] + 1
        end if
        Count_F ⇐ Count_F + 1
    end for
end for

// Phase 2: Weight assignment for each k-mer among the first M sampling quality score lines.
for q in F do
    S(q) = F(q)/Count_F;
end for

// Phase 3: Obtain Max_Weight among sampling lines in store for subsequent normalization.
Max_Weight ⇐ 0  // Max_Weight is used to normalize the weight for each line in Q^N.
for i = 1 to M do
    Line_Weight ⇐ 0  // Line_Weight is used to record the weight for each line in Q^N.
    len = length(Q_i)
    for j = 1 to (len − k + 1) do
        Line_Weight = Line_Weight + S(Q_i[j, j + k − 1])
    end for
    Line_Weight ⇐ Line_Weight/(len − k + 1)
    if Max_Weight ≤ Line_Weight then
        Max_Weight ⇐ Line_Weight
    end if
end for

// Phase 4: Calculate the total weight for each quality score line in Q^N
//          And partition them into two streams based on predefined threhold α.
for i = 1 to N do
    Line_Weight ⇐ 0
    len = length(Q_i)
    for j = 1 to (len − k + 1) do
        Line_Weight ⇐ Line_Weight + S(Q_i[j, j + k − 1])
    end for
    Line_Weight ⇐ Line_Weight/(len − k + 1)
    if Line_Weight/Max_Weight ≥ α then
        C_0 ⇐ Q_i
    else
        C_1 ⇐ Q_i
    end if
end for
```
