# Supplementary material for: LCQS: an efficient lossless compression tool of quality scores with random access functionality
Source: BMC Bioinformatics. 2020 Mar 18;21:109. doi: 10.1186/s12859-020-3428-7 (PMC7079445; doi:10.1186/s12859-020-3428-7)
Supplement: Supplementary file 2 — Additional file 2 Implementation details of adaptive k-mer packing method. [file 12859_2020_3428_MOESM2_ESM.pdf]

Implementation Details of Adaptive k-mer Packing Method

a) Get quality score Q_i : If Q_i equals to C, go to step (d); Else if Q_i belongs to [(C-3), C], go to step (b); Else if Q_i belongs to [(C-7), C], go to step (c); otherwise, map Q_i to (Q_i-32) and set i as i+1, and then go to step (a).

b) Get the next quality score Q_{i+1} : If Q_{i+1} belongs to [(C-3), C], go to step (e) ; Else if Q_{i+1} belongs to [(C-7), C], map $Q_i Q_{i+1}$ to ((Q_i-(C-7))*8 + (Q_{i+1}-(C-7)) +73) and set i as i+2 and go to step (a); Otherwise map Q_i to (Q_i-32) and set i as i+1, and then go to step (a).

c) Get the next quality score Q_{i+1} : If Q_{i+1} belongs to [(C-7), C], map $Q_i Q_{i+1}$ to ((Q_i-(C-7))*8 + (Q_{i+1}-(C-7)) +73) and set i as i+2 and go to step (a); Otherwise map Q_i to (Q_i-32) and set i as i+1, and then go to step (a).

d) Get the next quality score Q_{i+k}($k \leq 55$) until Q does not equal to C anymore. Record k. Map $Q_i Q_{i+1} Q_{i+2} Q_{...} Q_{i+k-1}$ to (k+200) and set i as i+k, and then go to step (a).

e) Get the next quality score Q_{i+2}: If Q_{i+2} belongs to [(C-3), C], map $Q_i Q_{i+1} Q_{i+2}$ to ((Q_i -(C-3))*16 + (Q_{i+1} -(C-3))*4 +(Q_{i+2} –(C-3)) + 137) and set i as i+3, and then go to step (a); Otherwise map $Q_i Q_{i+1}$ to ((Q_i-(C-7))*8 + (Q_{i+1}-(C-7)) +73) and set i as i+2 , and then go to step (a).
